# Comparative analysis of deep learning and radiomics models in predicting hepatocellular carcinoma differentiation via ultrasound

**DOI:** 10.3389/fmed.2025.1685725

**Published:** 2025-09-26

**Authors:** Luohang Xu, Yanhua Huang, Hong Fu, Jianhua Yu, Baochun Lu, Yalan Zheng, Jiaxuan Qian, Hongwei Qian

**Affiliations:** ^1^School of Medicine, Shaoxing University, Shaoxing, Zhejiang, China; ^2^Department of Ultrasound, Shaoxing People's Hospital, Shaoxing, China; ^3^Department of Hepatobiliary and Pancreatic Surgery, Shaoxing People's Hospital, Shaoxing, China; ^4^Shaoxing Key Laboratory of Minimally Invasive Abdominal Surgery and Precise Treatment of Tumor, Shaoxing, China

**Keywords:** hepatocellular carcinoma, radiomics, deep learning, ultrasound, tumor differentiation

## Abstract

**Objective:**

This study aimed to develop and compare predictive models for hepatocellular carcinoma (HCC) differentiation using ultrasound-based radiomics and deep learning, and to evaluate the clinical utility of a combined model.

**Methods:**

Radiomics and deep learning models were constructed from grayscale ultrasound images. A combined model integrating both approaches was developed. Model performance was assessed using receiver operating characteristic (ROC) curves, calibration curves, and decision curve analysis (DCA). Sensitivity, specificity, accuracy, and area under the curve (AUC) were compared, and statistical significance was evaluated with the DeLong test.

**Results:**

The radiomics model achieved an AUC of 0.736 (95% CI: 0.578–0.893), while the deep learning model achieved an AUC of 0.861 (95% CI: 0.75–0.972). The combined model outperformed both, with an AUC of 0.918 (95% CI: 0.836–1.0). The DeLong test indicated a significant improvement of the combined model over the radiomics model. Calibration analysis and the Hosmer–Lemeshow test showed good agreement between predictions and outcomes (*p* = 0.889). DCA demonstrated a higher net clinical benefit for the combined model across a range of thresholds.

**Conclusion:**

Integrating radiomics and deep learning enhances the predictive accuracy of ultrasound-based models for HCC differentiation, providing a promising non-invasive approach for preoperative evaluation.

## 1 Introduction

Hepatocellular carcinoma (HCC) is one of the most common types of tumors worldwide, particularly prevalent in Asia and sub-Saharan Africa, where incidence and mortality rates are exceptionally high (1–3). Despite significant advancements in medical technology over the past decades, the overall prognosis for HCC remains poor due to frequent late-stage diagnoses, limited therapeutic options, and a lack of reliable prognostic factors for predicting anti-tumor efficacy (4, 5).

Against this backdrop, the accurate preoperative prediction of HCC’s biological characteristics becomes critically important. A deeper understanding of the biological behavior of liver cancer can assist clinicians in developing more personalized treatment plans, optimizing therapeutic outcomes, extending patient survival, and enhancing quality of life. Therefore, developing new predictive tools and models, especially those that can accurately identify tumor biological characteristics at an early stage, has become a crucial direction in current liver cancer research.

Differentiation in HCC plays a crucial role in the clinical assessment and management of the disease. The degree of tumor differentiation is indicative of the tumor cells’ resemblance to normal hepatocytes, impacting their biological behavior and aggressiveness (6, 7). Poorly differentiated tumors are often associated with a higher degree of malignancy, more aggressive growth, and a greater propensity for invasion and metastasis (8). Consequently, assessing the differentiation status of HCC can provide valuable prognostic information, influence therapeutic strategies, and guide the selection of treatment modalities (9). Thus, accurate evaluation of tumor differentiation is essential for predicting clinical outcomes and optimizing patient care in HCC management.

Ultrasound imaging holds distinct advantages in the diagnosis and management of HCC, primarily due to its non-invasive nature, real-time imaging capabilities, and widespread availability. It provides a safe and cost-effective method for routine monitoring and assessment of liver lesions, allowing for repeated examinations without the risks associated with ionizing radiation (10). Radiomics and deep learning are two advanced methodologies that have shown substantial promise in the medical imaging field. Radiomics involves extracting a large number of features from medical images, which can be analyzed to reveal disease characteristics that are not visible to the naked eye (11). This approach has been used to develop predictive models that can assist in diagnosis, prognostication, and treatment planning (12, 13). On the other hand, deep learning, particularly convolutional neural networks, has the capability to automatically learn optimal features for tasks such as classification and segmentation directly from the image data, offering potentially higher accuracy and efficiency in image analysis (14).

Although both radiomics and deep learning have demonstrated significant advantages in various studies, most existing research has primarily focused on computed tomography (CT) and magnetic resonance imaging (MRI). For instance, Xia et al. (15) conducted a large-scale study involving 773 patients using CT-based radiomics, achieving an AUC of 0.86. In contrast, grayscale ultrasound is a more accessible and frequently used imaging modality in clinical settings due to its affordability and convenience. However, predictive modeling based on ultrasound remains underexplored. Furthermore, there is ongoing debate regarding the comparative performance of radiomics and deep learning (16, 17). Radiomics often shows stable performance, especially in small datasets, while deep learning requires larger samples to fully exploit its feature-learning capabilities (18). Feng et al. (19) demonstrated that deep learning models excel in large-scale imaging data. However, Du et al. (20) found radiomics to perform better in smaller-sample tasks. And the potential synergistic advantages of combining radiomics and deep learning in enhancing diagnostic accuracy and predictive performance in HCC have yet to be clearly defined.

The purpose of our study is to address these gaps by developing and evaluating predictive models for HCC differentiation based on ultrasound imaging using both radiomics and deep learning methodologies. Specifically, we aim to construct separate radiomics and deep learning models, and compare their diagnostic efficacy in predicting the differentiation status of HCC. Furthermore, we seek to create a combined model that integrates both approaches to determine if this hybrid model can outperform the individual methods. By focusing on ultrasound-based imaging, we intend to leverage its clinical advantages and explore its full potential in non-invasively predicting tumor biology.

## 2 Materials and methods

### 2.1 Study population

We retrospectively analyzed 224 patients diagnosed with hepatocellular carcinoma (HCC) who underwent surgery between September 2019 and April 2024. Patients were divided into well-differentiated (w-HCC) and moderately to poorly differentiated (mp-HCC) groups based on postoperative pathology. Inclusion criteria were: (1) age ≥ 18 years; (2) pathologically confirmed HCC; (3) ultrasound within 1 week prior to surgery; (4) complete clinical and pathological data; and (5) patient consent. Exclusion criteria included prior anti-tumor therapy, non-HCC pathology, other malignancies, poor image quality, and incomplete data.

**Figure 1:**
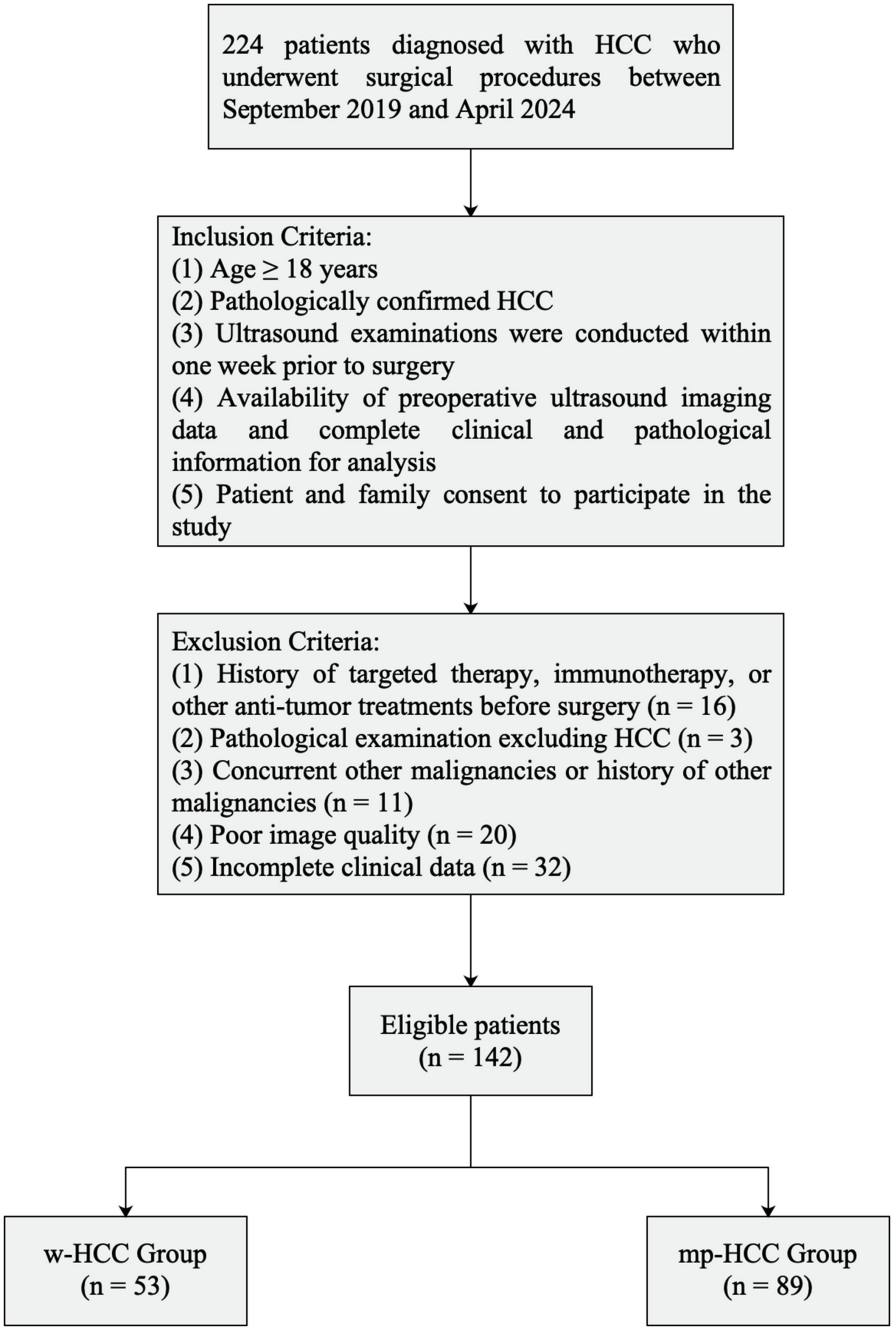
Inclusion/exclusion criteria flowchart.

The study was approved by the institutional review board, and informed consent was obtained. Patient selection is depicted in Figure 1.

### 2.2 Ultrasound procedure

Experienced radiologists conducted standardized grayscale ultrasound examinations. Patients were scanned in supine or lateral decubitus positions with transducers applied using coupling gel. Multiple views were acquired and stored in DICOM format. Equipment details are provided in the Supplementary material.

### 2.3 Histological and immunohistochemistry

Tumor specimens were processed from formalin-fixed, paraffin-embedded tissues. Hematoxylin and eosin (HE) staining evaluated morphology, and two pathologists independently classified differentiation status according to the 2019 WHO criteria, blinded to clinical outcomes (21).

### 2.4 Region of interest delineation

**Figure 2:**
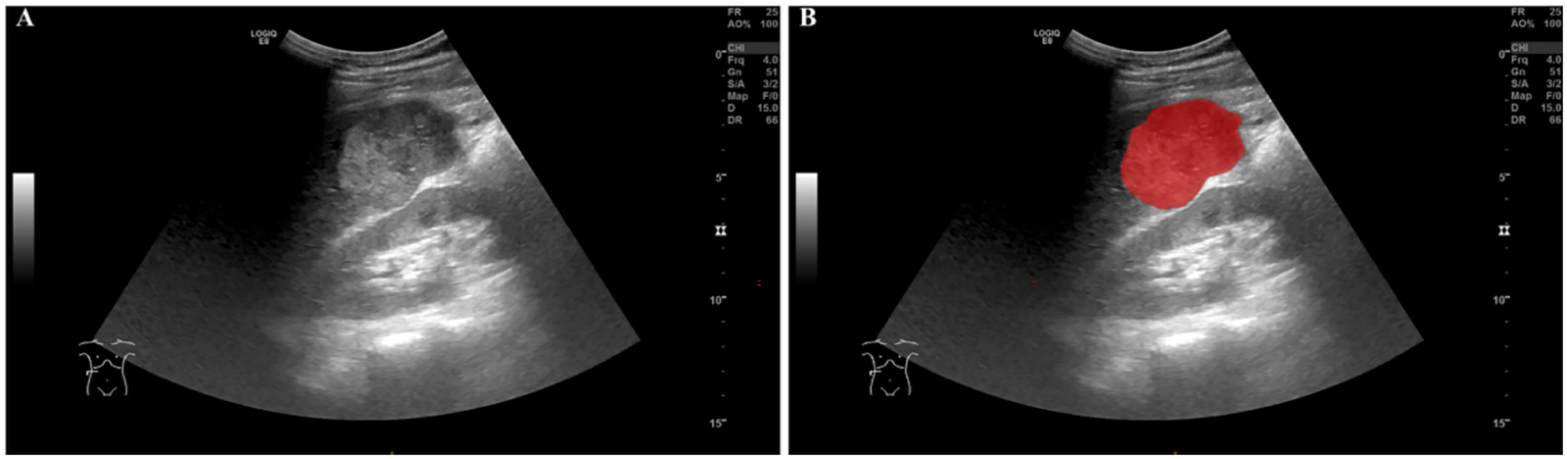
An example of delineating the region of interest (ROI) on abdominal ultrasound imaging using the ITK-SNAP software. **(A)** shows the original ultrasound scan, while **(B)** illustrates the ROI highlighted in red for analysis.

To ensure the accuracy and reproducibility of our radiomics analysis, the regions of interest (ROIs) were meticulously delineated on ultrasound images. Two experienced ultrasound radiologists (with 11 and 16 years of experience in abdominal ultrasound, respectively) independently performed the ROI delineation using the ITK-SNAP software (Version 4.0.0, www.itksnap.org) (22), a widely recognized tool for medical image segmentation (Figure 2). Each radiologist performed the delineation process twice, with a one-week interval between sessions. Inter- and intra-observer reproducibility was assessed via intraclass correlation coefficients (ICCs) (23). Radiologists were blinded to clinical information.

### 2.5 Radiomics feature extraction

Before feature extraction, the ultrasound images underwent a rigorous normalization process to ensure consistency and reliability of the radiomic features. This standardization involved converting all images to a uniform format, resampling the images to achieve a consistent spatial resolution of 3 × 3 × 3 mm^3^, and normalizing intensity values to 32 gray levels using a scale of 255. A median filter was applied to reduce speckle noise while preserving edge details. Additionally, segmentation alignment ensured that the delineated ROIs accurately corresponded to anatomical structures across all images.

After matching the largest tumor cross-section with the ROI NiFTI images, radiomics features were extracted using the open-source toolkit PyRadiomics. Extracted features included shape, first-order statistics, and texture features. Image filters (wavelet, square, square root, logarithm, exponential, gradient, local binary patterns) were applied to generate additional features. All features were standardized using Z-score normalization.

### 2.6 Deep learning feature extraction

For the extraction of deep learning features, we utilized the ResNet-101 (24) architecture, a highly effective convolutional neural network known for its ability to capture complex patterns in image data. The selection of ResNet-101 was based on its proven capacity to extract rich feature representations in previous studies (25–27). The input images for deep learning were first cropped as rectangular patches corresponding to the minimal bounding rectangle enclosing each manually delineated ROI, and then resized to 224 × 224 pixels, with pixel intensity values normalized to the [0, 1] range. These preprocessed images were then input into a pre-trained ResNet-101 model, which had been fine-tuned to adapt to the characteristics of ultrasound images of our HCC cases. During fine-tuning, we unfroze the final residual block and the fully connected layer while keeping earlier layers frozen to retain generic visual features. The model was trained using a batch size of 16, a learning rate of 1 × 10^−4^, and the Adam optimizer, for up to 100 epochs with early stopping based on validation loss. Data augmentation techniques, including horizontal flipping, random rotation (±15°), and brightness adjustment, were applied to improve generalizability. Fivefold cross-validation was used throughout the training process. During feature extraction, we captured the output of the final fully connected layer, resulting in a 1,000-dimensional feature vector for each image. Instead of directly using ResNet-101 for end-to-end binary classification, we treated it as a feature extractor to enable a consistent workflow with radiomics, where both types of features underwent the same dimensionality reduction and classification process. This approach also helped reduce the risk of overfitting in our relatively small dataset and improved the interpretability of selected features. To ensure consistency in subsequent analyses, the deep learning features were standardized using Z-score normalization. This hybrid strategy of deep feature extraction followed by classical machine learning has also been reported in previous studies as being more robust in small-sample settings compared with end-to-end deep learning (28, 29).

### 2.7 Feature dimension reduction and model construction

To ensure reproducibility of image segmentation, inter- and intra-observer consistency was evaluated using ICCs, retaining features with ICC > 0.8. Features with high multicollinearity (r > 0.8) were excluded. Subsequently, *t*-tests identified features significantly associated with tumor differentiation, followed by feature selection using the least absolute shrinkage and selection operator (LASSO).

Multiple classifiers—including Support Vector Machine, Random Forest, K-Nearest Neighbor, Logistic Regression, Decision Tree, Multilayer Perceptron, AdaBoost, Gradient Boosting, and XGBoost—were tested to build predictive models. A two-stage hyperparameter tuning process was employed, starting with RandomizedSearchCV for broad optimization, followed by GridSearchCV for refinement; both functions are part of the scikit-learn package (version 1.2.2) in Python. Fivefold cross-validation was used throughout. The model achieving the highest area under the curve (AUC) in the test set was selected as the optimal predictor. The AUC quantifies the overall ability of the model to discriminate between classes and is defined as the probability that a randomly chosen positive instance is ranked higher than a randomly chosen negative one.

### 2.8 Combined model construction

To construct the combined model, we used logistic regression to integrate the predicted probabilities from both the radiomics and deep learning models, leveraging the strengths of each individual model. The combined model’s performance was evaluated based on its AUC in the test set, and it was compared to the individual performances of the radiomics and deep learning models.

### 2.9 Comparison of image focus areas between radiomics and deep learning models

To compare the areas of focus between radiomics and deep learning (ResNet-101), we generated visualizations for both methodologies. For radiomics, we generated grayscale heatmaps to visualize the pixel intensity distribution within the tumor region. For the deep learning model, we applied Grad-CAM to the last convolutional block of the fine-tuned ResNet-101, computing the gradients of the target class score with respect to its feature maps to produce Class Activation Mapping (CAM) heatmaps that highlight the most discriminative regions within the ROI (30).

### 2.10 Statistical analysis

All radiomics procedures and statistical analyses were performed using Python 3.11. Continuous variables were compared with *t*-tests or Mann–Whitney U tests, categorical variables with chi-square or Fisher’s exact tests. Model performance was assessed using AUC, DeLong tests (31), calibration curves (32), and Decision curve analysis (DCA) (33). Statistical significance was determined using a two-sided *p*-value (denoted as p), with *p* < 0.05 indicating significance unless otherwise specified.

**Figure 3:**
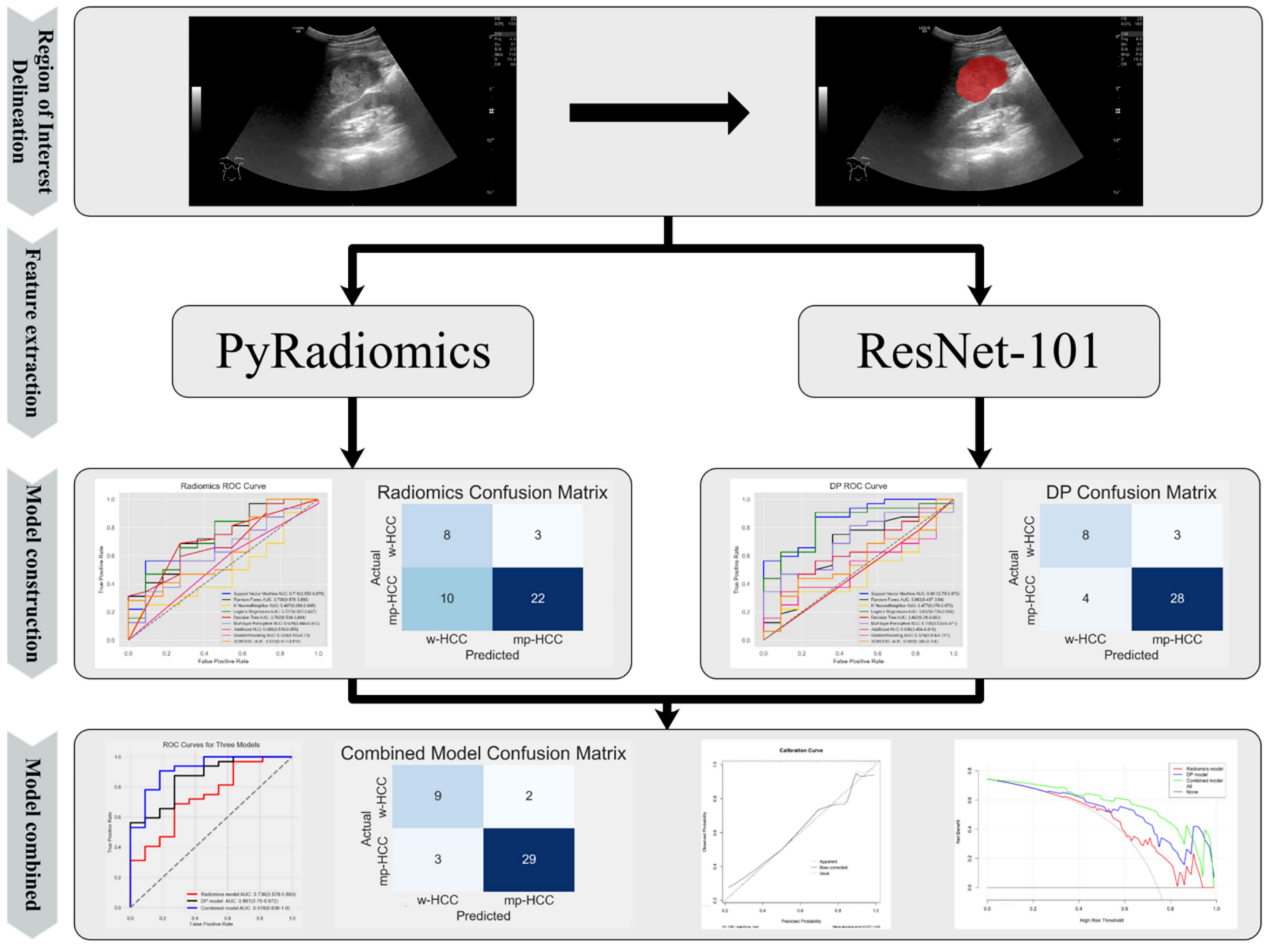
Schematic diagram of the process for radiomics and deep learning models.

The complete workflow is shown in Figure 3.

## 3 Results

### 3.1 Characteristics of the study population

We finally included a total of 142 patients in our study, who were grouped according to the differentiation degree of their tumors based on pathology: 53 in the w-HCC group and 89 in the mp-HCC group. The patients were randomly divided into training and testing groups in a 7:3 ratio, resulting in 99 patients in the training group and 43 patients in the testing group. Detailed clinical information is listed in Table 1. Tumor size showed a significant difference between the w-HCC and mp-HCC groups (4.0 cm [2.3–5.0] vs. 4.5 cm [2.5–6.6], *p* = 0.027); however, no statistical difference was observed in the training or testing groups. Other clinical parameters did not show significant differences across the various groups.

**Table 1:** Comparison of clinical characteristics between the well-differentiated HCC group (w-HCC) and the moderately-differentiated and poorly differentiated HCC group (mp-HCC).

Variables	w-HCC (*n* = 53)	mp-HCC (*n* = 89)	*p*	Training group (*n* = 99)		*p*	Testing group (*n* = 43)		*p*
				w-HCC (*n* = 42)	mp-HCC (*n* = 57)		w-HCC (*n* = 11)	mp-HCC (*n* = 32)	
Age (year)	66.96 ± 9.76	63.56 ± 10.54	0.06	66.38 ± 9.65	62.7 ± 11.42	0.098	69.18 ± 9.88	65.09 ± 8.53	0.206
AFP (mg/mL)	755.61 ± 4708.13	2337.73 ± 9823.17	0.277	945.71 ± 5272.24	2119.19 ± 6486.17	0.344	29.77 ± 72.35	2727.01 ± 13899.69	0.533
ALT (IU/L)	45.2 ± 52.56	38.7 ± 33.32	0.372	46.36 ± 52.31	40.34 ± 38.46	0.515	40.76 ± 53.27	35.78 ± 20.97	0.67
AST (IU/L)	47.19 ± 49.32	44.34 ± 41.79	0.716	44.78 ± 41.71	47.46 ± 50.51	0.781	56.4 ± 70.49	38.77 ± 16.31	0.206
TBIL (μmol/L)	18.32 ± 10.6	17.72 ± 17.73	0.826	18.1 ± 11.07	18.46 ± 21.08	0.92	19.14 ± 8.49	16.4 ± 8.92	0.39
DBIL (μmol/L)	5.67 ± 4.58	6.22 ± 10.26	0.715	5.13 ± 3.51	6.65 ± 12.36	0.444	7.75 ± 6.95	5.46 ± 4.46	0.227
Alb (g/L)	38.47 ± 4.21	39.3 ± 3.68	0.226	38.13 ± 4.26	39.46 ± 4.01	0.118	39.79 ± 3.75	39.01 ± 2.99	0.499
PT (s)	13.03 ± 1.51	12.8 ± 1.22	0.326	12.98 ± 1.59	12.7 ± 1.04	0.301	13.23 ± 1.17	12.98 ± 1.49	0.621
INR	1.06 ± 0.15	1.04 ± 0.09	0.187	1.06 ± 0.16	1.03 ± 0.08	0.243	1.08 ± 0.09	1.05 ± 0.11	0.402
Tumor Size (cm)	4.0 (2.3–5.0)	4.5 (2.5–6.6)	0.027*	4.09 ± 2.38	4.56 ± 2.79	0.393	3.92 ± 1.22	5.99 ± 3.56	0.072
Sex			0.1			0.208			0.263
Female	7	22		6	14		1	8	
Male	46	67		36	43		10	24	
HBsAg			0.516			0.448			0.957
Negative	17	24		14	15		3	9	
Positive	36	65		28	42		8	23	
Cirrhosis			0.726			0.579			0.922
Absent	24	43		19	29		5	14	
Present	29	46		23	28		6	18	
Multifocality			0.432			0.513			0.957
Absent	44	69		36	46		8	23	
Present	9	20		6	11		3	9	

HCC, hepatocellular carcinoma; w-HCC, well-differentiated HCC group; mp-HCC, moderately-differentiated and poorly differentiated HCC group; AFP, alpha fetoprotein; ALB, albumin level; ALT, alanine aminotransferase; AST, aspartate aminotransferase; TBIL, total bilirubin; DBIL, directed bilirubin; PT, prothrombin time; INR, international normalized ratio; *, *p* < 0.05.

### 3.2 Feature selection

In this study, we extracted a total of 1,411 radiomics features from the ultrasound images. After applying intra-ICC analysis, 1,366 features were retained. Subsequent inter-ICC analysis further reduced the number of features to 1,343. To address multicollinearity, features with a correlation coefficient greater than 0.8 were eliminated. Further feature selection was conducted using *t*-tests and LASSO regression, resulting in four radiomics features being included in the study. These features and their corresponding coefficients are summarized in Table 2. The detailed LASSO selection process is illustrated in Supplementary Figure S1.

**Table 2:** The selected radiomics features and their coefficient values.

Filter	Feature class	Feature	Coefficient
Original	firstorder	Maximum	−0.089122
Wavelet-LHL	glszm	ZoneEntropy	−0.005337
Logarithm	glszm	SizeZoneNonUniformity	−0.101838
lbp-2D	glrlm	RunLengthNonUniformity	−0.071497

For the deep learning features, we utilized a locally fine-tuned ResNet-101 model to extract 1,000 features from the ultrasound images. We then applied the same feature reduction methodology—addressing multicollinearity, performing *t*-tests, and employing LASSO regression. This process reduced the number of deep learning features to 12, which were subsequently included in the study. The LASSO regression results for deep learning feature selection are also provided in Supplementary Figure S2.

Given the imbalance in sample sizes between the w-HCC and mp-HCC groups, we employed the Synthetic Minority Over-sampling Technique (SMOTE) (34) to balance the sample sizes between the two groups within the training set, which was done before feature dimensionality reduction and selection. By applying SMOTE on the training data, we ensured that the training set was balanced, thereby enhancing the robustness and reliability of our predictive models during the training phase.

### 3.3 Model construction

We employed various modeling techniques to identify the optimal algorithm for our predictive models. Optimal model parameters were selected using RandomizedSearchCV followed by GridSearchCV for precise tuning (details provided in the Supplementary material).

**Figure 4:**
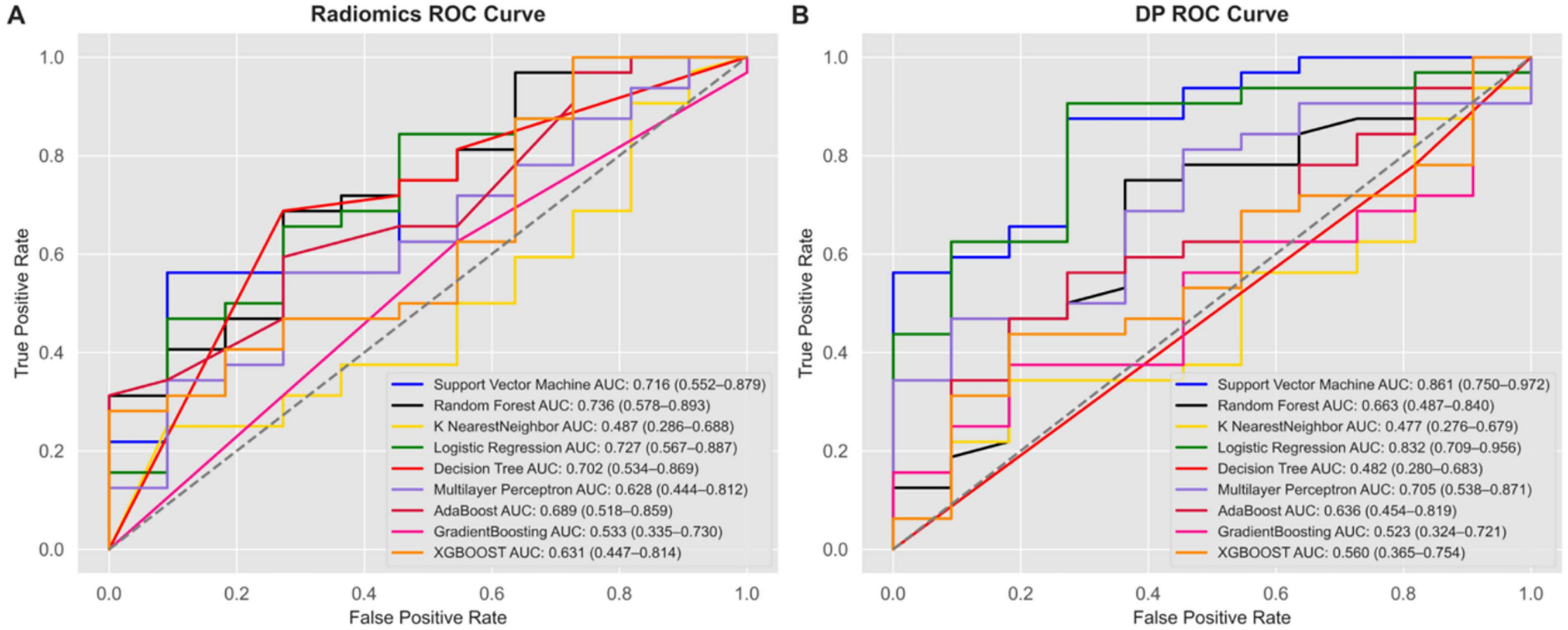
Receiver operating characteristic (ROC) curves illustrating the diagnostic performance of different modeling methods in radiomics and deep learning models. In the radiomics model **(A)**, the random forest algorithm achieved the highest diagnostic performance with an AUC of 0.736 (95% CI: 0.578–0.893). In the deep learning model **(B)**, the support vector machine demonstrated the highest diagnostic performance with an AUC of 0.861 (95% CI: 0.75–0.972). The best-performing algorithms—Random Forest for radiomics **(A)** and Support Vector Machine for deep learning **(B)**—were selected for subsequent model comparison and fusion.

**Figure 5:**
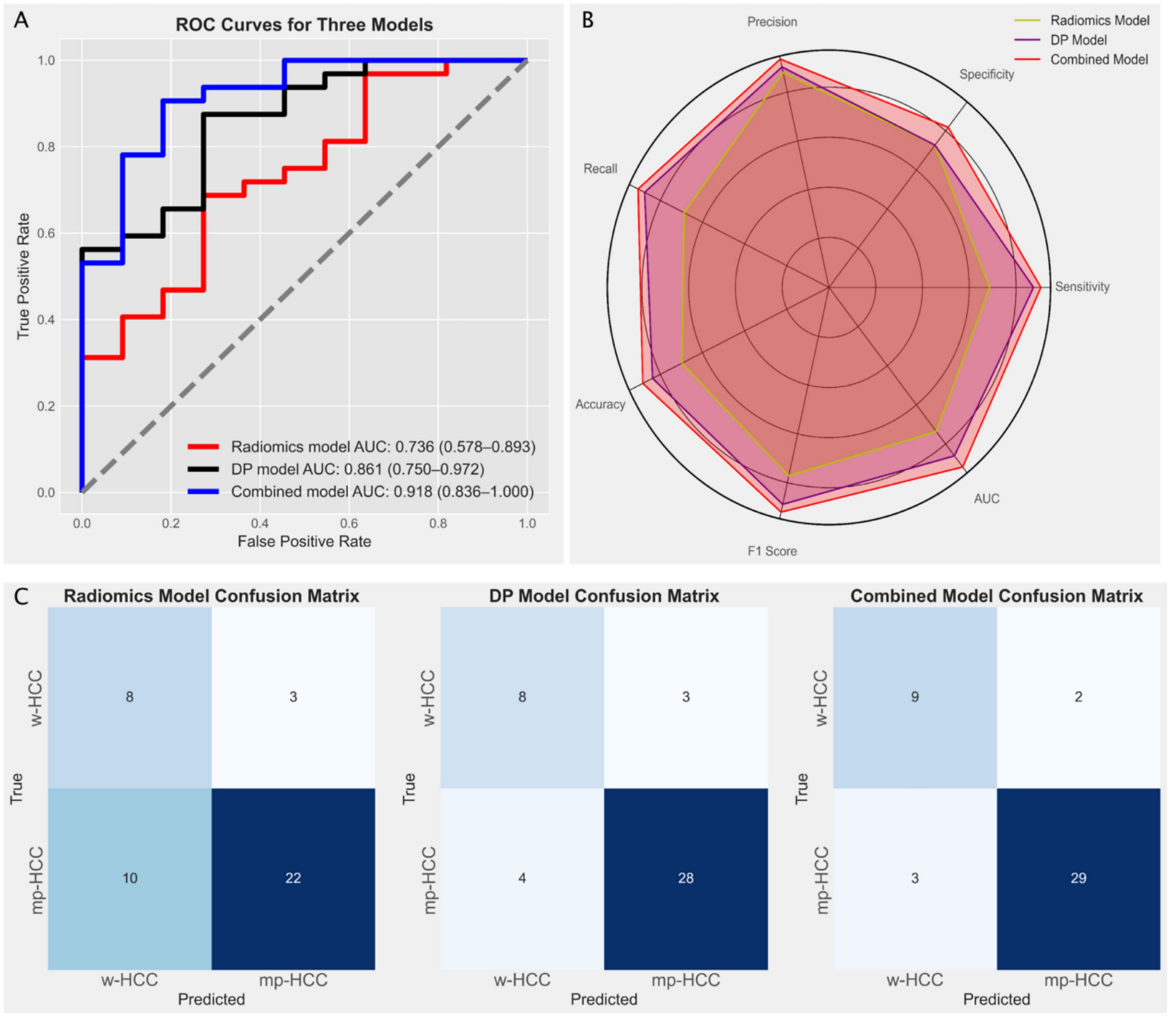
**(A)** Receiver operating characteristic (ROC) curves illustrating the diagnostic performance of three models: the radiomics model (red line, based on Random Forest) with an AUC of 0.736 (95% CI: 0.578–0.893), the deep learning model (black line, based on Support Vector Machine) with an AUC of 0.861 (95% CI: 0.75–0.972), and the combined model (blue line) with the highest AUC of 0.918 (95% CI: 0.836–1.0). **(B)** Radar chart comparing the performance metrics (precision, recall, specificity, F1 score, and accuracy) of the three models, indicating the superior performance of the combined model. **(C)** Confusion matrices for the radiomics model, the deep learning (DP) model, and the combined model, illustrating the distribution of true positives, false positives, true negatives, and false negatives in predicting well-differentiated HCC (w-HCC) and moderately to poorly differentiated HCC (mp-HCC).

In the radiomics model, the Random Forest (RF) algorithm achieved the highest AUC of 0.736 (95% CI: 0.578–0.893), while in the deep learning model, the Support Vector Machine (SVM) achieved the highest AUC of 0.836 (95% CI: 0.75–0.972) (Figure 4). The DeLong test indicated no significant difference between the AUCs of the radiomics and deep learning models (*p* = 0.224). Based on these results, we constructed a combined model using logistic regression to integrate the outputs of the best-performing radiomics and deep learning models. The combined model yielded the highest AUC of 0.918 (95% CI: 0.836–1.000) (Figure 5A). DeLong tests showed no significant difference between the deep learning and combined models (*p* = 0.384), but a significant improvement of the combined model over the radiomics model (*p* = 0.042), suggesting that radiomics and deep learning provide complementary predictive value. This added benefit may result from integrating global statistical features captured by radiomics with localized abstract patterns learned by deep learning.

To provide a more comprehensive comparison, we calculated additional performance metrics, including precision, recall, specificity, accuracy, and F1 score, and visualized them using radar charts (Figure 5B). The combined model showed consistent advantages across all metrics, especially in F1 score and accuracy, reflecting its superior balance between sensitivity and specificity. Confusion matrices (Figure 5C) further supported this, with fewer misclassifications observed in the combined model, particularly for mp-HCC cases. Detailed metric values including true positives (TP), false positives (FP), true negatives (TN), false negatives (FN), and AUC are summarized in Table 3.

**Table 3:** The performance of the radiomics, deep learning, and combined models.

Evaluation indicators	Radiomics model	Deep learning model	Combined model
TP ↑	22	28	**29**
FP ↓	3	3	**2**
FN ↓	10	4	**3**
TN ↑	8	8	**9**
AUC ↑	0.736	0.861	**0.918**
TPR (Recall) ↑	0.688	0.875	**0.906**
TNR ↑	0.727	0.727	**0.818**
Precision ↑	0.88	0.903	**0.935**
ACC ↑	0.698	0.837	**0.884**
F1 Score ↑	0.772	0.889	**0.921**

TP, True Positives; FP, False Positives; FN, False Negatives; TN, True Negatives; AUC, Area Under the Curve; TPR, True Positive Rate; TNR, True Negative Rate; ACC, Accuracy; ↑ indicates higher is better; ↓ indicates lower is better. Bold values indicate the best performance among the three models.

**Figure 6:**
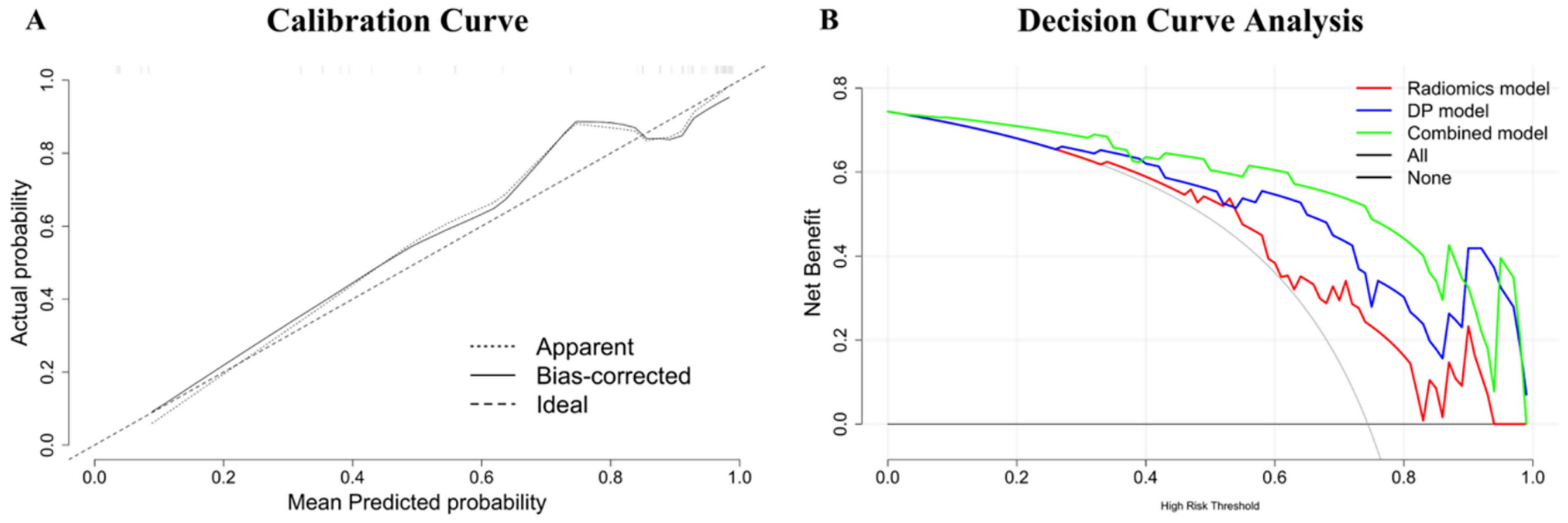
**(A)** Calibration curve of the combined model, demonstrating the agreement between predicted probabilities and observed outcomes. The curve shows the apparent, bias-corrected, and ideal lines, indicating the model’s accuracy and reliability in predicting HCC differentiation. **(B)** Decision Curve Analysis (DCA) for the radiomics model, deep learning (DP) model, and the combined model. The DCA curve illustrates the net benefit of each model across a range of threshold probabilities. The “All” and “None” lines represent two reference strategies: treating all patients (All) and treating none (None), respectively. The combined model demonstrates superior clinical utility compared to the individual models across most threshold ranges.

Calibration analysis showed good agreement between predicted and observed outcomes, as assessed by the Hosmer–Lemeshow test (*p* = 0.889) (32), supporting the model’s reliability (Figure 6A). Decision curve analysis (DCA) further confirmed that the combined model offers superior clinical utility across a wide range of decision thresholds, suggesting greater potential for aiding preoperative decision-making (Figure 6B). To facilitate clinical interpretation, we further constructed a nomogram based on the combined model, which is provided in Supplementary Figure S3.

### 3.4 Comparison of image focus areas between radiomics and deep learning models

To explore differences in focus between radiomics and deep learning (ResNet-101), we generated visualizations based on the same manually segmented ROI. For radiomics, a grayscale heatmap of pixel intensities within the ROI was created, illustrating the underlying distribution of signal values. These raw intensity patterns are uniformly processed by PyRadiomics to extract global features (e.g., mean, standard deviation, energy, entropy), without assigning differing importance to subregions.

In contrast, for the deep learning model, a CAM was produced, revealing spatially localized areas that had the greatest influence on model predictions. Unlike radiomics, which treats all pixels within the ROI equally, ResNet-101 adaptively focuses on more discriminative subregions, effectively applying spatial attention within the ROI.

**Figure 7:**
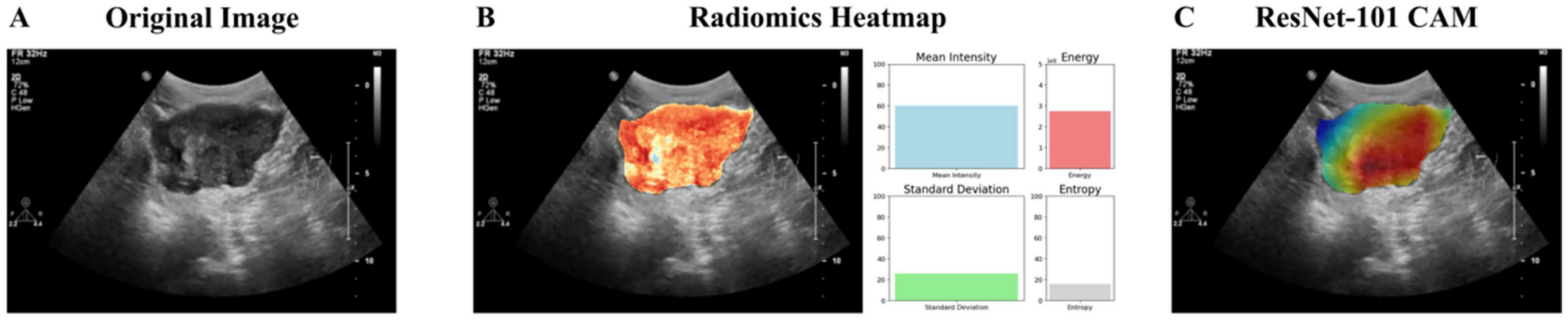
Comparison of the image focus areas between radiomics and deep learning (ResNet-101) models. **(A)** The original ultrasound image of the tumor region. **(B)** Radiomics heatmap displaying pixel intensity distribution within the manually defined ROI; PyRadiomics computes global features from the entire region without localized weighting. **(C)** Class Activation Map (CAM) generated by the ResNet-101 model, illustrating spatially localized attention to subregions within the same ROI that most influenced the classification outcome.

Figure 7 shows a representative example, comparing the grayscale radiomics heatmap and the CAM, which together highlight the difference in how each model interprets the same tumor region.

## 4 Discussion

In this study, we developed and compared ultrasound-based radiomics and deep learning models for predicting HCC differentiation, and further constructed a combined model integrating both approaches. Both individual models showed good predictive performance, with the combined model achieving the best results. Our findings highlight the advantage of integrating diverse methodologies to comprehensively capture and analyze information from medical images. Moreover, our findings suggest that grayscale ultrasound, when combined with advanced analytical techniques, holds substantial promise for the non-invasive prediction of HCC biological characteristics—addressing a notable gap in the literature compared to CT and MRI-based studies.

While both radiomics and deep learning models showed good diagnostic performance, it is important to consider the influence of dataset size. With limited data, the generalization ability of deep learning models may be restricted, impacting their diagnostic performance. This limitation is particularly important in the medical field and can affect the applicability of deep learning models in certain clinical scenarios. In our study, with a sample size of 146, we revealed that the AUC of the deep learning model was higher than that of the radiomics model. However, the Delong test indicated no statistically significant difference between the two (*p* = 0.224). This suggests that, even though deep learning models may exhibit higher diagnostic efficacy, their advantage is not always statistically significant, especially in smaller sample sizes. This finding aligns with other studies that emphasize the stability and efficiency of radiomics models in small datasets (35).

The four selected radiomic features primarily reflect intensity and texture heterogeneity, which are closely related to tumor differentiation. Two features—ZoneEntropy and SizeZoneNonUniformity from the GLSZM—capture the randomness and variability of homogeneous intensity zones, suggesting greater tissue disorganization in poorly differentiated HCC (36). The RunLengthNonUniformity from the GLRLM describes irregularity in gray-level runs, reflecting subtle texture disruption (37). In contrast, Maximum intensity reflects local hotspots such as necrosis or hypervascular regions (38). Together, these features characterize both structural irregularity and microenvironmental complexity relevant to HCC grading.

Although tumor size differed between groups in the overall cohort, no significant difference was observed within the training or testing subsets. This further supports the notion that conventional clinical variables may have limited predictive value for HCC biological characteristics, as shown in previous studies including our own (39, 40). When we combined the radiomics and deep learning models, the resulting hybrid model achieved superior performance, with higher AUC, sensitivity, specificity, and accuracy than either model alone. This combined model showed a statistically significant improvement over the radiomics model. The enhancement likely stems from the complementary strengths of both methodologies: radiomics provides detailed, quantitative image features, while deep learning offers high-level, abstract feature recognition (28, 29). In our study, the comparison of image focus areas between radiomics and deep learning models further supports this finding. As shown in the heatmaps (Figure 7), the visual comparison of the heatmap and CAM demonstrates the distinct but complementary focus areas of the two approaches. Radiomics provides a broad overview that can identify extensive variations within the tumor, while deep learning captures the intricate details and patterns that may be indicative of tumor differentiation.

Further evaluation of the combined model using calibration curves and DCA underscores its clinical utility and reliability. The calibration curve indicated that the combined model had good agreement between predicted probabilities and observed outcomes, with a Hosmer-Lemeshow test *p*-value of 0.889, signifying good calibration. The DCA curve demonstrated that the combined model offers a higher net benefit across a range of threshold probabilities compared to the individual radiomics and deep learning models. This indicates that the combined model not only provides better diagnostic performance but also has practical applicability in clinical decision-making. This integration not only improves predictive accuracy but also offers a more comprehensive understanding of the tumor’s biological characteristics, supporting more informed clinical decision-making (41).

Despite the promising results, our study has several limitations. The retrospective nature of the analysis may introduce selection bias, and the single-center study design limits the generalizability of our findings. Additionally, the relatively small sample size could affect the robustness and external validity of the models. To address the limitations of a modest dataset, we avoided end-to-end deep learning classification and instead used a feature-extraction–dimension-reduction–modeling workflow, which is more robust in small samples and allowed a fair comparison with radiomics. Another limitation is that we did not compare the performance of different deep learning architectures, which might have provided further insights into the optimal backbone choice for ultrasound-based prediction. Future studies should aim to validate our models in larger, multi-center cohorts, and systematically evaluate multiple architectures to enhance their external validity.

## 5 Conclusion

In conclusion, our study highlights the potential of combining radiomics and deep learning methodologies to enhance the predictive accuracy of ultrasound-based models for HCC differentiation. The promising results underscore the need for further research to validate and refine these models, paving the way for their implementation in clinical practice to improve the management and outcomes of patients with HCC.

## Data Availability

The original contributions presented in the study are included in the article/Supplementary material, further inquiries can be directed to the corresponding author.
