# Supplementary material for: Comparative analysis of deep learning and radiomics models in predicting hepatocellular carcinoma differentiation via ultrasound
Source: Front Med (Lausanne). 2025 Sep 26;12:1685725. doi: 10.3389/fmed.2025.1685725 (PMC12511121; doi:10.3389/fmed.2025.1685725)
Supplement: Supplementary file 1 [file Supplementary_file_1.docx]

**Supplementary material**

**S1 -Ultrasound machines**

Ultrasound examination was performed by using one of the following ultrasound machines: LOGIQ E8 (GE Healthcare, United States; C5-1 convex array probes, 1–5 MHz); LOGIQ E9 (GE Healthcare, United States; C5-1 convex array probes, 1–5 MHz); Aplio 500 (Toshiba Medical systems, Japan; 6C1 probe, 1–6 MHz); i800 (Cannon Medical systems Corporation, Japan; i8CX1 probe, 1-8MHz); and Resona 7T (Mindray, China; SC6-1 U probe, 1-6MHz).

**Supplementary Figures**


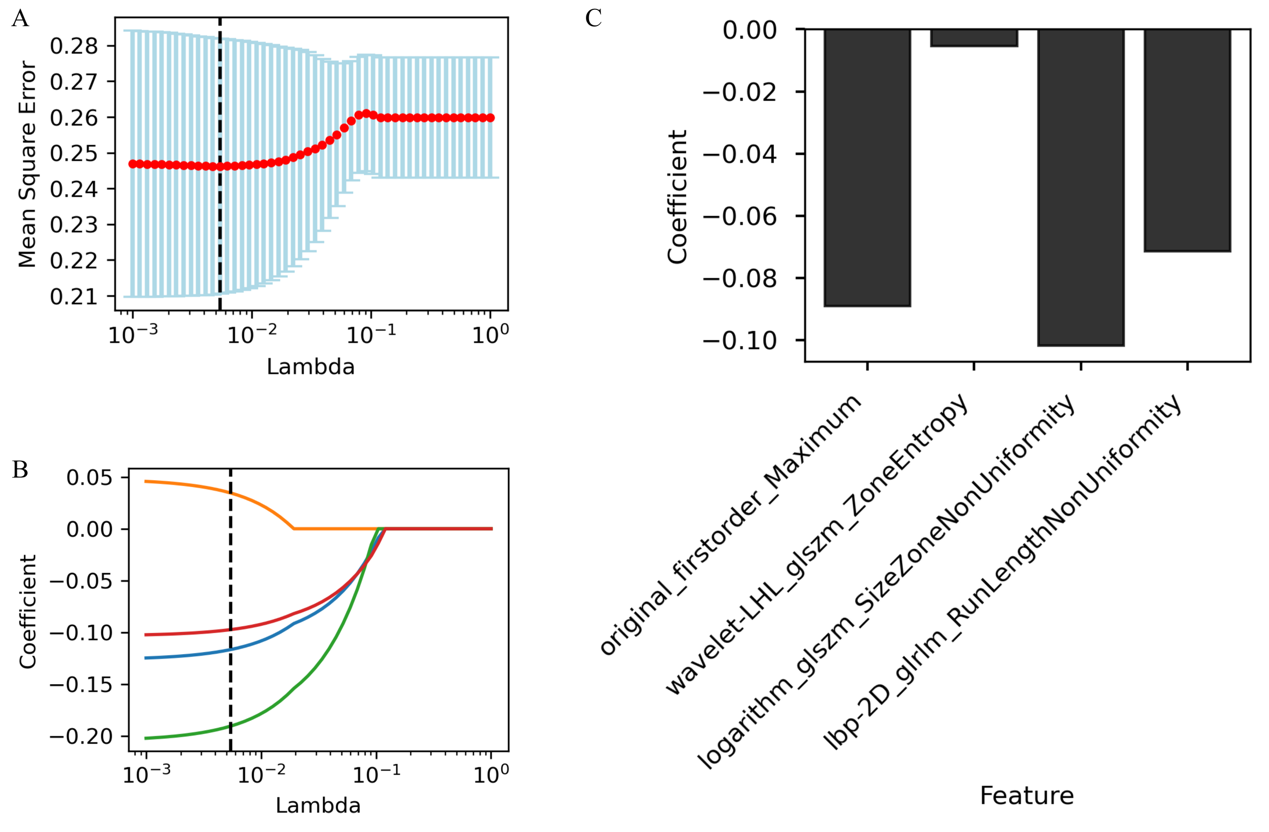


**Figure S1.** LASSO feature selection process for radiomics features. (A, B) Least absolute shrinkage and selection operator (LASSO) regression complexity was controlled using a tuning parameter lambda; the optimal lambda value for the minimized mean squared error was 0.005428675439323859. (C) The 4 selected variables and their coefficient values are shown.


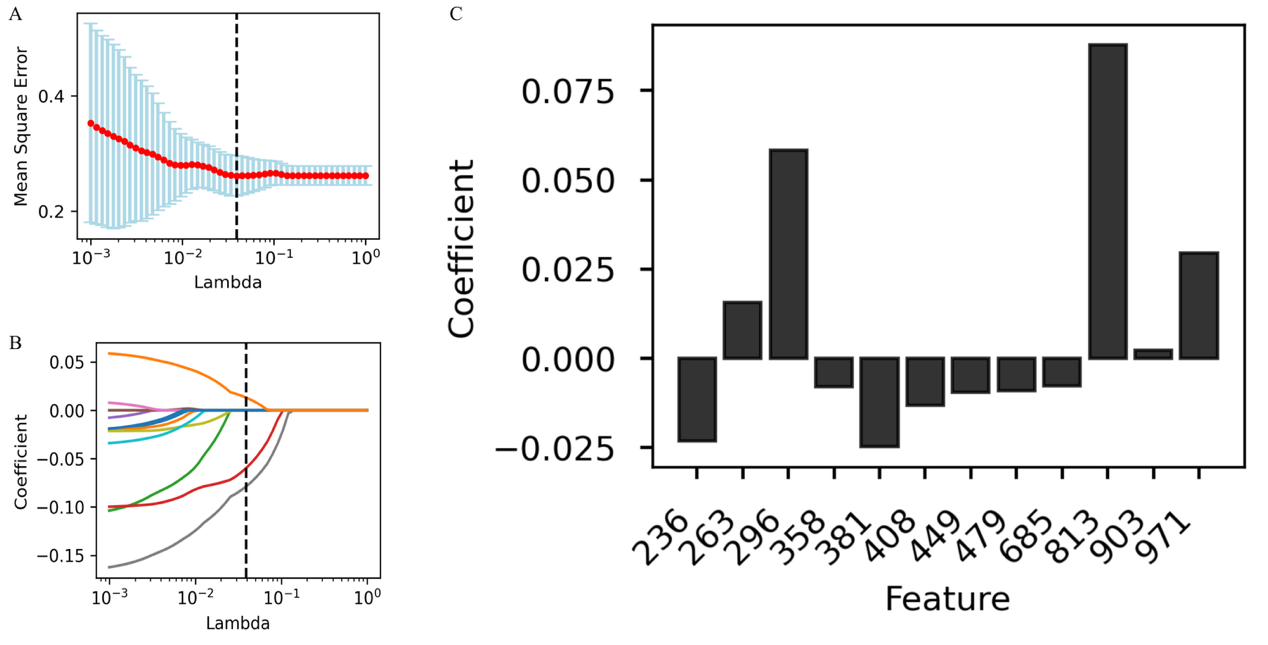


**Figure S2.** LASSO feature selection process for deep learning (ResNet-101) features. (A, B) Least absolute shrinkage and selection operator (LASSO) regression complexity was controlled using a tuning parameter lambda; the optimal lambda value for the minimized mean squared error was 0.03906939937054617. (C) The 12 selected variables and their coefficient values are shown.


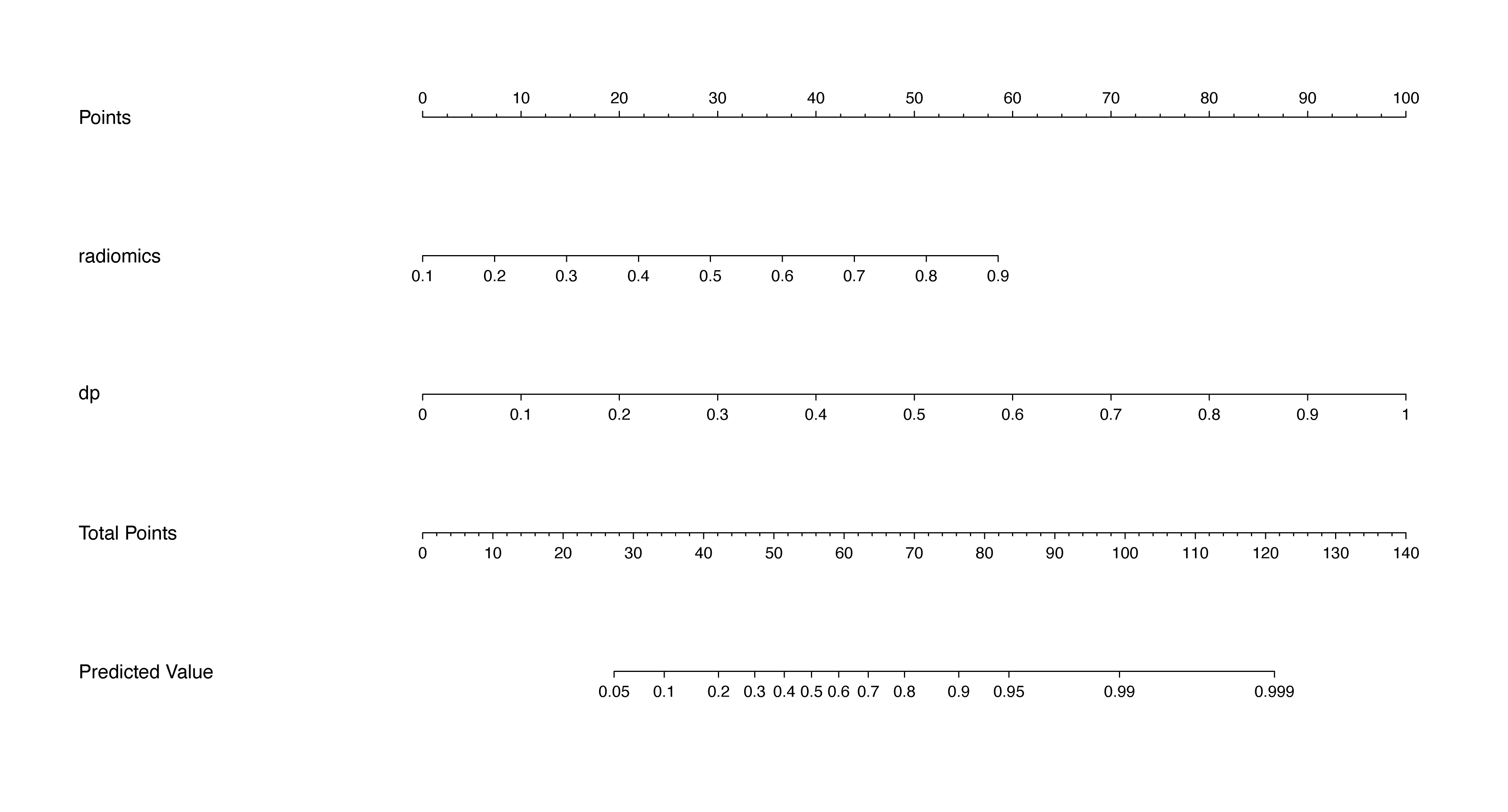


**Figure S3.** Nomogram constructed based on the combined model integrating radiomics and deep learning models.

**Supplementary Tables**

**Table S1**

**Best parameters in Radiomics Model**

| **Model** | **Best Parameters** |
| --- | --- |
| SVM | {'C': 26.120029033345595, 'gamma': 916.8737656057649, 'kernel': 'sigmoid'} |
| RandomForest | {'max_depth': 10, 'min_samples_split': 10, 'n_estimators': 250} |
| KNN | {'n_neighbors': 3, 'p': 1, 'weights': 'distance'} |
| LogisticRegression | {'C': 0.007, 'penalty': 'l2'} |
| DecisionTree | {'max_depth': 20, 'min_samples_split': 5} |
| MLPClassifier | {'activation': 'relu', 'alpha': 0.001, 'hidden_layer_sizes': (50, 50), 'learning_rate': 'invscaling'} |
| AdaBoostClassifier | {'learning_rate': 0.01, 'n_estimators': 250} |
| GradientBoostingClassifier | {'learning_rate': 1000.0, 'max_depth': 3, 'n_estimators': 100} |
| XGBOOST | {'gamma': 0.1, 'learning_rate': 1.0, 'max_depth': 3, 'n_estimators': 150} |

**Table S2**

**Best parameters in Deep learning Model**

| **Model** | **Best GridSearch Parameters** |
| --- | --- |
| SVM | {'C': 244.3600760310954, 'gamma': 0.0146677575115858, 'kernel': 'sigmoid'} |
| RandomForest | {'max_depth': 30, 'min_samples_split': 2, 'n_estimators': 350} |
| KNN | {'n_neighbors': 19, 'p': 2, 'weights': 'distance'} |
| LogisticRegression | {'C': 0.025, 'penalty': 'l2'} |
| DecisionTree | {'max_depth': 30, 'min_samples_split': 2} |
| MLPClassifier | {'activation': 'relu', 'alpha': 3.181818181818181e-06, 'hidden_layer_sizes': (50, 50), 'learning_rate': 'invscaling'} |
| AdaBoostClassifier | {'learning_rate': 1.0, 'n_estimators': 200} |
| GradientBoostingClassifier | {'learning_rate': 0.1, 'max_depth': 5, 'n_estimators': 150} |
| XGBOOST | {'gamma': 0.001, 'learning_rate': 1.0, 'max_depth': 5, 'n_estimators': 100} |

**Table S3**

**Best parameter in Combined Model**

| **Model** | **Best GridSearch Parameters** |
| --- | --- |
| LogisticRegression | {'C': 0.2, 'penalty': 'l2'} |
